# Machine Learning Approach to Forecast Chemotherapy-Induced Haematological Toxicities in Patients with Rhabdomyosarcoma

**DOI:** 10.3390/cancers12071944

**Published:** 2020-07-17

**Authors:** Vesna Cuplov, Nicolas André

**Affiliations:** 1 SMARTc, Marseille Cancer Research Center (CRCM), UMR Inserm 1068, CNRS UMR 7258, Aix Marseille Université U105, Institut Paoli Calmettes & APHM, 13385 Marseille, France; 2 Paediatric Haematology and Oncology Department, La Timone Children’s Hospital, AP-HM, 13385 Marseille, France; nicolas.andre@ap-hm.fr

**Keywords:** children, rhabdomyosarcoma, haematological toxicity, neutropenia, thrombocytopenia, machine learning, artificial intelligence

## Abstract

Developing precision medicine is a major trend in clinical oncology. The main adverse effects of ifosfamide, actinomycin D and vincristine (IVA) treatment for rhabdomyosarcoma are haematological toxicities such as neutropenia or thrombocytopenia. The severity of these effects vary among patients but their dynamic profiles are similar. A non-empirical adjustment of the chemotherapy dose to avoid severe toxicities could help secure the treatment administration. Twenty-four patients with rhabdomyosarcoma treated with IVA chemotherapy courses were selected. Before and during each cycle, routine multiple blood cell counts were performed allowing for a dynamic study of the haematological toxicities. We developed a machine learning analysis using a gradient boosting regression technique to forecast the ifosfamide induced haematological toxicities as a function of neutrophils and platelets initial levels and the initial ifosfamide dose. To validate models’ accuracy, predicted and observed neutrophils and platelets levels were compared. The model was able to reproduce the dynamic profiles of the haematological toxicities. Among all cycles, the mean absolute errors between predicted and observed neutrophils and platelets levels were 1.0 and 72.8 G/L, respectively. Adjusting a patient’s ifosfamide dose based upon the predicted haematological toxicity levels at the end of a treatment cycle could enable tailored treatment.

## 1. Introduction

Rhabdomyosarcoma (RMS) is an aggressive and highly malignant form of sarcoma [1,2]. RMS is the most common soft tissue sarcoma in children and adolescents. RMS staging and risk grouping combines: age at diagnosis, size, and localization of the primary tumor, existence of metastatic lymph nodes and/or distant visceral or bone metastasis. Additional risk factors are complete resection and PAX/FOX01 fusion gene status [3]. The principal treatment includes surgery for complete tumor removal, radiation therapy and systemic chemotherapy because of the high risk of metastasis. The main chemotherapy drugs used to treat patients with intermediate and high risk RMS are vincristine, actinomycin D, ifosfamide, etoposide, irinotecan, and doxorubicin [4]. In Europe, the most commonly administered combination is ifosfamide, actinomycin D, and vincristine (IVA) [5,6]. Ifosfamide is an anti-neoplasic drug which acts by direct interaction on the DNA and inhibits the transcription and replication of the RNA. Principal adverse effects of ifosfamide are renal and urologic toxicity and neutropenia. Vincristine is an anti-mitotic cancer drug which blocks the cancer cell in metaphase and triggers its apoptosis. The principal adverse effect of vincristine is peripheral neuropathy. Actinomycin D is an antibiotic that inhibits the action of topoisomerase II, which is a type of enzyme that controls the changes in DNA structure inhibiting the activity of the cell. This drug inhibits the transcription of the DNA to RNA. The principal adverse effects of actinomycin D are haematological and digestive toxicities. The IVA protocol is administered every 21 days. The IVA protocol is active against a wide range of paediatric tumors, such as RMS and Ewing sarcomas. This treatment induces haematological toxicities, mainly neutropenia and thrombocytopenia. When not directly life-threatening, these toxicities impair the efficacy of the treatment, because they result in either reducing the chemotherapy dose or treatment discontinuation to allow the body to recover from these toxicities.

Building statistical models to describe the dynamics of blood cells upon IVA administration is appealing but challenging. Several modeling-based strategies have been developed over the last two decades to describe the chemotherapy-induced haematological toxicity, including both mathematical modeling [7,8,9] and machine learning (ML). However, these two approaches are two distinct paradigms.

Mathematical modeling of chemotherapy-induced myelosuppression requires full quantitative representation of the main biological processes associated with haematopoiesis, such as the understanding of the chemotherapeutic drugs effects on the bone marrow [10,11,12]. Using a semi-physiologic population pharmacokinetic-pharmacodynamic model to estimate the haematopoietic toxicity of carboplatin, Schmitt et al. proposed an optimal carboplatin target area under the curve as a function of pretreatment or concomitantly administered chemotherapies [13]. A model proposed by Fornari et al. mimics the neutrophils maturation chain based on five compartments [14].

On the other hand, machine learning focuses on how computers learn from data while using efficient algorithms—this implies that all relevant information must be included in the dataset. Artificial neural networks were implemented by Pella et al. to predict acute toxicity in organs at risk following prostate radiotherapy [15]. In their work, Shibahara et al. have developed a machine learning approach to estimate nimustine hydrochloride induced myelosuppression through analysis of patient blood cell counts before treatment [16]. In another study, Gunčar et al. have built two classifier models that predict haematologic disease while using a machine learning random forest algorithm and based on laboratory blood test results [17]. Oyaga-Iriarte et al. implemented several machine learning classification algorithms that predict irinotecan toxicity grades in metastatic colorectal cancer while using demographic data, liver function tests, and tumor markers, combined with pharmacokinetic parameters [18]. Machine learning can also be used for other purposes such as in depth analysis of medical imaging [19,20,21]. In their work, Zacharaki et al. have developed a computer-assisted classification method that is based on support vector machines combining conventional MRI and perfusion MRI for distinguishing different types of brain tumors [22].

Unlike mathematical modeling, the ML approach does not require in-depth understanding of the modeled phenomenon or process. In addition, a variety of machine learning algorithms are available as open source tools and libraries, such as the scikit-learn library in Python [23]. For these reasons, the main objective of this work is to develop a machine learning model that would predict patients’ haematological toxicity in time, during an IVA chemotherapy cycle. Predicting the patient’s level of toxicity at the end of a cycle should allow clinicians to adapt the initial chemotherapy dose and avoid treatment delays due to severe neutropenia and/or thrombocytopenia. These models have been integrated in a web-based application, which could be used by clinicians to guide their decisions regarding chemotherapy dose adaptation.

## 2. Materials and Methods

### 2.1. Patients and Treatment Protocol

Patients selected in this retrospective study had histologically confirmed rhabdomyosarcoma. They were previously untreated, except for initial surgery/biopsy within eight weeks after the diagnosis. All twenty-four patients that were selected in this study were treated in the Paediatric Haematology and Oncology Department of La Timone Children’s Hospital in Marseille between 1995 and 2019. Selected patients had a minimum, maximum, and median age of 0.4, 15.4, and 5.5 years. They received up to nine cycles of IVA chemotherapy courses, as described in Table 1, which possesses a non-mandatory part. Indeed, for the two first cycles of chemotherapy, vincristine was also given at day 8 and day 15. All patients from this study have been perfused with ifosfamide with the same protocol schema. Both parents systematically signed an informed consent. The children were also proposed to sign informed consent and would do so depending on their age and will to give consent. The Institutional Ethics Committee from the Marseille Public University Hospital System (Assistance Publique Hôpitaux de Marseille) approved the retrospective study.

### 2.2. Data Organization

**Figure 1:**
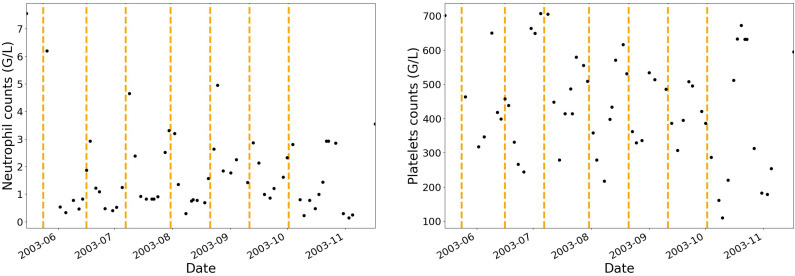
Example of one patient’s neutrophils and platelets data over the course of seven IVA treatment cycles. Black dots represent the observed data (neutrophils and platelets blood cell counts). Vertical dashed lines show the beginning dates of each treatment cycle.

Administration protocols and haematological blood cell counts (i.e., neutrophils, platelets, monocytes, lymphocytes and leucocytes) have been collected for twenty-four patients. None of the patients have been treated with granulocyte-colony stimulating factor. Before and during each IVA cycle, multiple blood cell counts were performed allowing for a dynamic analysis of the haematological toxicities in patients as shown in Figure 1 where the vertical dashed lines separate each treatment cycle.

The original data have been re-arranged in a tabular data structure with labeled axes for further manipulation, where each row corresponds to the patient’s result of a blood cell count at a particular date of the treatment. Due to multiple blood cell counts in each IVA cycle and the shape of the haematological toxicity profiles, the data have been interpolated at all additional days (dates) of the treatment where no blood tests have been performed in order to provide additional insight into the learning problem for supervised learning models. Common interpolation methods were tested, such as linear, polynomial, spline, and piecewise cubic hermite interpolating polynomial [24]. The data (observed data plus additional data from interpolation) were finally grouped into individual dataframes, each corresponding to an IVA cycle number. Several information were added in the re-arranged dataset, such as the neutrophils and platelets initial values (measured at the beginning of each cycle), the patient’s weight, the ifosfamide dose normalized to the patient’s weight, and the number of days that has elapsed from the start of the cycle (i.e., cycle elapsed time in days, which is between 1 and 21). The neutrophils and platelets initial values were obtained either directly from the original data or computed at the cycle start date using the interpolation function (sometimes the blood test at the beginning of the cycle was performed one or two days prior to the chemotherapy treatment).

### 2.3. Regression Algorithms and Training Datasets

Since vincristine has no haematological toxicity and given the low haematological toxicity of actinomycin D in comparison to ifosfamide, only the ifosfamide induced haematological toxicity was modeled. The neutrophils and platelets levels were modeled at any time during a treatment cycle using the following features: initial values of neutrophils and platelets, the initial value of the ifosfamide dose normalized to the patient’s weight and the elapsed time in days in the cycle. To develop these models, we applied a machine learning technique on the interpolation-based resampled training dataset that corresponds to an IVA cycle. There is one training dataset per cycle that groups equivalent patients: the training dataset of cycle *c* contains all patients who underwent all *c* successive cycles. For the first four treatment cycles, the number of equivalent patients in the training datasets is 20, 12, 9, and 8, respectively, resulting in 431, 256, 210, and 197 haematological observations or instances after interpolation-based resampling. From the initial 24 patients, only 20 were included in the training dataset for cycle 1, because either a part of the haematological observations or the IVA doses were missing and could not be fully retrieved retrospectively.

In order to build the neutrophils and platelets predictive models, several regression techniques were tried, such as regularized linear models (Lasso, Ridge) and gradient boosted decision trees. Regularization techniques are used to deal with overfitting. Lasso (Ridge) regularization shrinks the regression coefficients by penalizing the regression model with a term called L1-norm (L2-norm), which is the sum of the absolute (squared) coefficients. For both of the methods, the constant that multiplies the penalty term and the maximum number of iterations were optimized using a grid search technique with the mean squared error as the scoring parameter that defines the model evaluation rules. On the other hand, the gradient boosting technique produces a composite model that combines the efforts of multiple weak prediction models (typically decision trees) to make a strong model. Each additional weak model reduces the mean squared error of the overall model. This boosting technique starts with a simple model $f_{0}\left(x\right)$ that gives an initial approximation of the target *y* (e.g., neutrophils level) given the patient’s feature *x* (e.g., dose normalized to the patient’s weight) and gradually goes towards the known target *y* by adding one or more adjustments $\Delta_{m}\left(x\right)$:

$$\begin{array}{ccc} y & = & f_{0}\left(x\right)+\sum_{m=1}^{M}\Delta_{m}\left(x\right) \end{array}$$

The gradient boosting regressor parameters are tree and boosting specific. Table 2 shows the list of parameters that were optimized while using the same technique as for Lasso and Ridge regressors. The input of each of these cycle-based regressors is a vector composed of the patient’s initial levels of neutrophils and platelets, the ifosfamide dose normalized to the patient’s weight and the cycle elapsed time in days. The output of the regressors is a scalar: neutrophils or platelets count. During the gradient boosting model’s parameters optimization, we applied the special process of early stopping, which monitors the model’s performance on a testing dataset (validation fraction was set to 0.3) and stops the training procedure once the performance on the testing dataset does not improve beyond a certain number of iterations. This technique avoids overfitting by finding the least number of iterations, which is sufficient to build a model that generalizes well to unseen data. Prediction performances (coefficients of determination $R^{2}$) were compared in order to choose between Lasso, Ridge, and gradient boosting regression algorithms.

### 2.4. Model Validation

To evaluate the performance of neutrophils and platelets estimators on new data, we tested them following a 10-fold cross validation procedure at the observation level. In addition, we performed a leave-one-out cross validation, where the regressor is trained on all od the evaluable data, except one patient, and a prediction is made for that patient. The training datasets are specific to the IVA cycle. Each validation was performed while using a set of optimized regression parameters.

The absolute errors between predicted and true haematological toxicity values were compared. For each patient (left out of the training dataset), the predicted neutrophils and platelets profiles were compared to the true profiles. Prediction intervals (PI) were provided as a way to quantify the accuracy of the predictions:

$$\begin{array}{ccc} \mathrm{PI}(\alpha ) & = & \alpha \times \frac{1}{N-2}\sqrt{\sum_{m=1}^{N}{\left(y_{m}^{\mathrm{True}}-y_{m}^{\mathrm{Pred}}\right)}^{2}} \end{array}$$

with *N* the number of observables (number of patients in the training dataset) and α being equal to 1.64 and 1.96 for a significance level of 90% and 95%, respectively.

## 3. Results

**Figure 2:**
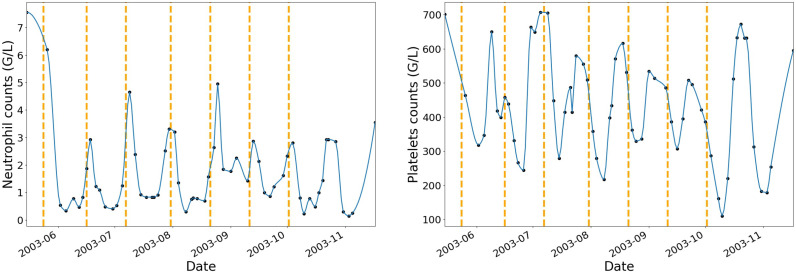
Example of the piecewise cubic hermite interpolating polynomial function applied on one patient’s neutrophils and platelets data. The blue curve shows the interpolation that was used to extract additional blood cell counts when no blood tests have been performed. Black dots represent the observed data. Vertical dashed lines show the beginning dates of each treatment cycle.

To begin with, neutrophils and platelets data have been interpolated at additional dates where no blood tests have been performed while using a piecewise cubic hermite interpolating polynomial. Figure 2 shows an example of one patient’s neutrophils and platelets profiles after interpolation with vertical dashed lines that correspond to the beginning of each IVA treatment cycle.

The choice of the interpolating function was based on the review of the haematological toxicity dynamic profiles after interpolation. The piecewise cubic hermite interpolating polynomial (pchip) method gave more natural curves on the interpolated values when compared to linear, polynomial, or spline of order 2 and 3. For each chemotherapy cycle, the pchip interpolation augmentation method allowed for increasing the original dataset by 70%.

Subsequently, regularized linear and gradient boosting regressors were trained on the interpolated resampled data. To avoid overfitting, the number of iterations for each regressor was optimized by finding the least number of iterations, which is sufficient for building a model that generalizes well to unseen data. The performances of Lasso, Ridge, and gradient boosting models are summarized in Table 3, where the mean prediction performances ($R^{2}$) across the three models are shown along with the difference of $R^{2}$ in the training and testing datasets following a 10-fold cross validation procedure at the observation level. For the first four treatment cycles, optimized gradient boosting regressors yielded mean $R^{2}$ values that were greater than 0.74 for neutrophils and 0.84 for platelets. The mean differences of $R^{2}$ values in the training and testing data were smaller than 0.17 and 0.12 for neutrophils and platelets, respectively. We found that the gradient boosting model was associated to the highest coefficient of determination on randomly split training and testing samples.

Accordingly to this result and for the first four cycles of the IVA treatment, neutrophils and platelets dynamic profiles have been modeled using cycle specific training datasets and optimized gradient boosting regression parameters.

**Figure 3:**
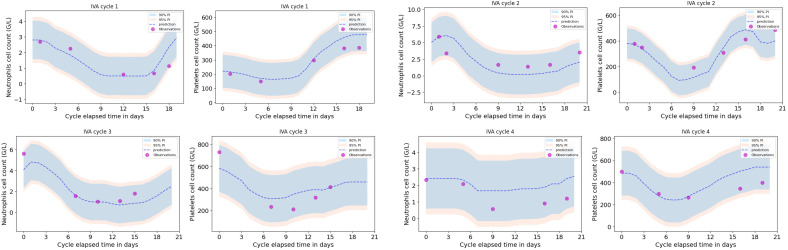
Examples of one patient’s validation results obtained for the neutrophils and platelets prediction models of the four first IVA treatment cycles. The magenta dots are the (true) observed neutrophils and platelets counts from the patient’s blood tests. The validation patient’s data were removed from the dataset on which the regression models were trained and optimized. The dashed line shows the neutrophils and platelets predictions from the trained models with input parameters that corresponded to the validation patient’s neutrophils and platelets initial counts and the ifosfamide initial dose. The shaded areas, which illustrate the 90% and 95% prediction intervals, are proportional to the cycle-based model’s standard deviation computed from the entire leave-one-out cross validation procedure.

A leave-one-out cross validation was performed, as shown in Figure 3 for one particular patient (the one removed from the training dataset) for the first four treatment cycles. Neutrophils and platelets dynamic profiles were compared to the predictions for that particular patient together with the 90% and 95% prediction intervals.

Table 4 summarizes all of the results of the leave-one-out cross validation in terms of the median value (over one cycle) together with the interquartile range of the error between predicted and true values of the blood cell counts ($y^{\mathrm{Pred}}-y^{\mathrm{True}}$) for each patient that was removed from the dataset on which the regression model was trained. A negative error illustrates that the model under-estimates the toxicity level.

Table 5 shows the mean absolute errors and associated standard deviations for each of the four first treatment cycles, computed from the leave-one-out cross validation results shown in Table 4. Among all four treatment cycles, the mean errors (in absolute value) between predicted and observed neutrophils and platelets levels are 1.0 and 72.8 G/L, respectively. Assuming that the absolute error between predicted and true haematological toxicity is a reasonable prediction evaluation metric, the magnitude of these errors give an idea of how good the predictions are for new patients. Among the first four cycles, the errors are small from the point of view of an oncologist reviewing neutrophil and platelet levels of patients under chemotherapy.

### Web-Based Application

**Figure 4:**
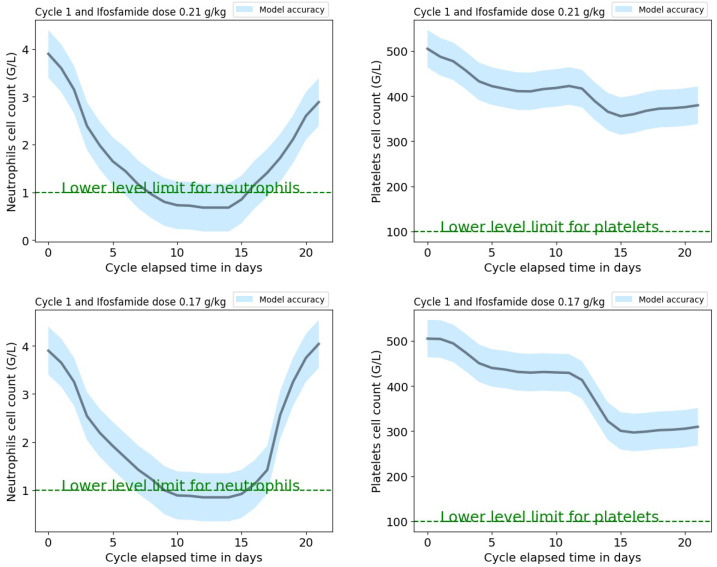
Example of a result obtained while using the web-based application. It shows the predicted neutrophils and platelets dynamic profiles for the first IVA treatment cycle for a new patient with initial neutrophils and platelets levels at 3.9 and 505 G/L and an ifosfamide weight-normalized dose set to 0.21 g/kg (upper plots) and 0.17 g/kg (lower plots). The blue bands illustrate the neutrophils and platelets model’s mean errors obtained from the validation procedure applied to the selected treatment cycle (Table 5). Green dashed lines show neutrophils and platelets lower limits for mild neutropenia and thrombocytopenia.

A web-based application has been developed with a Python 3.0 kernel and it will be available on the pythonanywhere platform. The application interface requires the following input parameters: the treatment cycle (between 1 and 4), the initial neutrophils and platelets cell counts, and the ifosfamide dose normalized to the patient’s weight from the protocol. Input parameters values should be within the range that corresponds to these parameters from the cycle-dependent dataset used to train the models. The application provides the predictions of the new patient’s neutrophils and platelets dynamic profiles, as shown in Figure 4 with the corresponding mean absolute error (which is cycle dependent) extracted from the validation procedure and shown in Table 5. These predictions could help the clinician to tailor the patient’s protocol by modifying the initial ifosfamide dose normalized to the patient’s weight or administer granulocyte growth factors to avoid unacceptably low levels of neutrophils or platelets at the end of an IVA cycle.

## 4. Discussion

In the attempt to anticipate chemotherapy induced haematologic toxicity, we have computed a machine learning based model that could accurately predict blood cell count dynamics in individual paediatric patients, with rhabdomyosarcoma receiving the IVA chemotherapy course.

The interpolation augmentation method that was used to resample the original data has some limitations, as it may be dependent on the number of observations per patient. The error on the additional observations where no blood tests have been performed would be larger for patients with less observation points. On average, the number of haematological observations per patient per cycle of 21 days is 6, which corresponds to a blood test every 3.5 days. We believe that the unimodal shape of the neutrophils or platelets profiles (aplasia usually followed by the recovery of normal blood counts) is an advantage for the interpolation procedure.

For consistency, the data used to compute the models were composed of patients who did not skip a cycle of treatment making sure that the haematological toxicity levels were not biased by recovered neutrophils and platelets levels due to a chemotherapy break. The size of the training dataset decreases with the cycle number, leading to larger errors on the toxicities predictions; therefore, both models must be updated and saved as new data are acquired.

A non-ML model, such as the autoregressive integrated moving average (ARIMA), could bring some interesting insights to our problem but is limited to a single time series, unless more complex models are pieced together. Informations about the initial levels of neutrophils or platelets and the ifosfamide dose cannot be easily integrated in an ARIMA model. With ML models, multiple series can be estimated within the same framework providing additional reason for the use of ML in this study. Indeed, seasonal and non-seasonal ARIMA models are powerful, as long as the model’s parameters are well configured.

Our neutrophil models are trained on patients who were not injected with growth factors (i.e., GCSF). The use of these growth factors changes the patient’s neutrophil profile; therefore, neutrophil level predictions for a new patient under GCSF might be under-estimated with our model. Patients demographics, such as age and gender, were not added as features in our models because these informations were not always known for each patient in this retrospective study. On the other hand, patient’s gender does not appear to be involved in the ifosfamide metabolism or toxicity [25]. In future work with additional data and an investigation to retrieve the missing information from the initial cohort, new covariates will be incorporated in our models, such as age, the use of granulocyte growth factors, and pharmacogenetic markers.

This work is a proof of concept that shows the potential use of neutropenia and thrombocytopenia predictions on ifosfamide dose adjustment for patients with significant reductions in neutrophil or platelet counts, which, if not anticipated, could cause a delay in their subsequent chemotherapy administrations and compromise their treatment outcome. With our ML models—which are available on our online application—neutrophil and platelet counts could be predicted over the course of a chemotherapy cycle based on different ifosfamide doses. This offers the possibility to adjust the ifosfamide dose, which enables personalized treatment of patients undergoing chemotherapy. Indeed, therapy related toxicities have been negatively associated with outcome and survival [26].

In the near future, we hope to collaborate with more institutions and convince them to provide and gather more data in order to update our predictive models and improve their accuracy. These updated models will be validated while using an independent cohort of patients with intermediate or high risk rhabdomyosarcoma and then tested prospectively through a clinical trial. The web application will greatly facilitate this testing in a multi-center setting. Once, these steps have been completed, the application could be proposed to a collaborative paediatric sarcoma group to test ifosfamide dose adjustment based on the prediction of our models. The models could also be further tested and calibrated on patients with Ewing sarcoma who also receive IVA courses.

### Data and Code Availability

The data that support the findings of this study are available on the public repository figshare upon reasonable request. The analysis was developed on a Jupyter notebook with a Python 3.0 kernel. A series of free and open source Python libraries were utilized, such as SciPy for scientific computing, scikit-learn for machine learning, and the multiprocessing module in order to make best use of all CPU cores [23]. The code for data processing, including interpolation, gradient boosting regression model parameters optimization, and validation, are available on the GitHub platform upon demand.

## 5. Conclusions

We used a machine learning technique with a gradient boosting regressor to model haematological toxicities in patients with rhabdomyosarcoma treated with several cycles of IVA chemotherapy. Our results show, with a good accuracy, that it is possible to forecast the neutrophils and platelets profiles for a new patient when providing the model with the neutrophils and platelets initial values together with the initial value of the ifosfamide dose normalized to the patient’s weight. This work could be extended to other chemotherapy treatments and/or pathologies.

## Figures and Tables

**Table 1:** The ifosfamide, actinomycin D, and vincristine (IVA) protocol schema. The text in italic emphasises the non mandatory part of the protocol: vincristine can be administered at day 8 and day 15 for the first two cycles. All patients from this study have been perfused with ifosfamide with the same protocol schema and for the two first cycles of chemotherapy, vincristine was also given at day 8 and day 15.

Schedule	Ifosfamide	Vincristine	Actinomycin D
Day 1	3 g/m${}^{2}$ (3 h perfusion)	1.5 mg/m${}^{2}$ (injection)	1.5 mg/m${}^{2}$ (injection)
Day 2	3 g/m${}^{2}$ (3 h perfusion)	-	-
Day 3	-	-	-
Day 8	-	*1.5 mg/m${}^{2}$ (injection)*	-
Day 15	-	*1.5 mg/m${}^{2}$ (injection)*	-

**Table 2:** Tree and boosting specific parameters of the gradient boosting regression algorithm as they appear in the scikit-learn package together with their definition. The last column shows the range of values that were tested in the algorithm optimization procedure.

	Definition	Range of Values
Tree specific parameters		
min_samples_split	Minimum number of samples in a	[2, 19]
	tree node to be considered for splitting	
min_samples_leaf	Minimum number of samples in a	[2, 19]
	tree terminal node	
max_depth	Maximum depth of a tree	[5, 15]
Boosting specific parameters		
learning_rate	Impact of each tree in the final outcome	[0.05, 0.2]
n_estimators	Number of trees to be modeled	[20, 80]
subsample	Fraction of samples to be randomly selected	0.8
	for each tree	

**Table 3:** Comparison between Lasso, Ridge and gradient boosting predictions for IVA cycles 1 to 4. The table shows the mean $R^{2}$ value for each model following a 10-fold cross validation procedure at the observation level. The value in parenthesis is the mean difference of $R^{2}$ values in the training and testing datasets (in absolute value).

Cycle	Neutrophils: $R_{\mathrm{test}}^{2}$ ($|R_{\mathrm{train}}^{2}-R_{\mathrm{test}}^{2}|$)			Platelets: $R_{\mathrm{test}}^{2}$ ($|R_{\mathrm{train}}^{2}-R_{\mathrm{test}}^{2}|$)		
	Lasso	Ridge	Grad. Boost.	Lasso	Ridge	Grad. Boost.
1	0.23 (0.00)	0.23 (0.01)	0.81 (0.11)	0.49 (0.01)	0.49 (0.01)	0.94 (0.04)
2	0.34 (0.03)	0.36 (0.02)	0.75 (0.14)	0.08 (0.06)	0.14 (0.08)	0.84 (0.12)
3	0.40 (0.13)	0.41 (0.13)	0.82 (0.10)	0.07 (0.08)	0.06 (0.08)	0.84 (0.10)
4	0.28 (0.03)	0.28 (0.02)	0.74 (0.17)	0.16 (0.02)	0.17 (0.02)	0.88 (0.08)

**Table 4:** The median value (over one cycle) of the error between prediction and observation for neutrophils and platelets levels for each patient that was not included in the dataset on which the regression model was trained and optimized. The value in parenthesis represents the median value of the interquartile range.

**Cycle 1**	001	002	003	004	005	006
Neutrophils	1.2 (1.6)	−0.6 (0.5)	0.1 (0.3)	−0.8 (2.0)	0.8 (0.4)	−0.3 (0.7)
Platelets	−58.6 (107.9)	−36.4 (216.8)	20.8 (61.8)	11.5 (85.1)	48.6 (29.7)	63.1 (185.1)
	007	008	009	010	011	012
Neutrophils	0.2 (2.0)	0.5 (0.4)	0.5 (0.6)	−0.1 (0.2)	0.0 (0.2)	−0.2 (1.1)
Platelets	−37.6 (128.3)	1.5 (29.9)	−3.6 (199.4)	59.9 (46.1)	−180.3 (239.1)	123.2 (55.4)
	014	016	019	020	021	022
Neutrophils	−2.1 (4.5)	0.8 (1.4)	0.7 (2.9)	0.1 (1.2)	0.8 (0.8)	−0.3 (4.4)
Platelets	−7.8 (22.0)	−42.9 (188.2)	−18.1 (55.9)	6.2 (37.0)	−18.6 (132.5)	−14.1 (78.9)
	023	024				
Neutrophils	−0.0 (0.4)	−0.2 (2.5)				
Platelets	−29.2 (144.6)	−49.3 (97.6)				
**Cycle 2**	001	002	004	005	006	007
Neutrophils	−0.1 (0.4)	3.8 (1.1)	−0.2 (1.0)	0.4 (1.1)	0.2 (1.2)	−0.1 (1.3)
Platelets	−130.1 (59.1)	−290.4 (279.6)	1.5 (84.3)	89.6 (126.2)	31.3 (139.0)	−39.6 (88.1)
	009	010	018	019	020	021
Neutrophils	−0.3 (2.0)	0.3 (0.4)	−1.2 (0.9)	−1.5 (2.0)	4.0 (1.1)	0.2 (0.4)
Platelets	−29.6 (43.9)	117.1 (113.8)	−6.2 (86.6)	153.0 (131.5)	49.5 (163.5)	206.0 (71.7)
**Cycle 3**	001	002	004	005	007	010
Neutrophils	−0.2 (1.0)	4.2 (6.4)	0.0 (0.9)	−0.2 (1.4)	−0.4 (0.9)	−0.6 (1.0)
Platelets	−117.3 (117.9)	−397.4 (311.4)	22.6 (101.0)	37.0 (261.3)	71.4 (75.0)	135.5 (311.0)
	019	020	021			
Neutrophils	−4.8 (8.8)	−0.1 (0.3)	1.8 (0.3)			
Platelets	123.5 (148.0)	43.1 (189.2)	89.2 (134.4)			
**Cycle 4**	001	002	003	005	007	018
Neutrophils	3.4 (1.2)	2.7 (1.1)	−0.2 (0.5)	1.4 (0.6)	1.0 (0.9)	−1.9 (6.7)
Platelets	−146.7 (191.2)	−38.8 (37.4)	45.3 (42.8)	41.5 (159.5)	8.6 (157.9)	75.6 (122.2)
	021	023				
Neutrophils	−0.2 (3.2)	−3.6 (7.5)			
Platelets	32.4 (128.7)	164.5 (195.4)				

**Table 5:** Mean absolute error and standard deviation for neutrophils and platelets models for the first four IVA treatment cycles. These errors are computed from patients’ median absolute errors over one cycle shown in Table 4.

Cycle	Mean Absolute Error (G/L)	
	Neutrophils: Mean (Std)	Platelets: Mean (Std)
1	0.52 (0.49)	41.57 (42.38)
2	1.03 (1.35)	95.32 (84.52)
3	1.37 (1.75)	115.22 (106.77)
4	1.80 (1.25)	69.18 (52.94)
